# Aptamer based high throughput colorimetric biosensor for detection of *staphylococcus aureus*

**DOI:** 10.1038/s41598-020-66105-7

**Published:** 2020-06-08

**Authors:** Tianxiao Yu, Hong Xu, Yan Zhao, Yanjie Han, Yao Zhang, Jingrui Zhang, Caihong Xu, Wenju Wang, Qing Guo, Jun Ge

**Affiliations:** Department of clinical laboratory, the fourth hospital of Shijiazhuang (Shijiazhuang gynaecology and obstetrics hospital), Shijiazhuang, China

**Keywords:** Biochemistry, Biotechnology

## Abstract

To develop a high throughput colorimetric biosensor for detection of *Staphylococcus aureus* (SA) based on specific aptamer and catalysis of dsDNA-SYBR Green I (SG I) complex. SA specific aptamer was immobilized on a 96-well plate by hybridization with the capture probe anchored on the plate surface through streptavidin-biotin binding. In presence of SA, the aptamer was dissociated from the capture probe-aptamer duplex due to the stronger interaction between the aptamer and SA. The consequent single-strand capture probe could be hybridized with a three-way junction (TWJ) probe. With the presence of SG I, the dsDNA-SG I complex catalyze the oxidation of 3,3′,5,5′-tetramethylbenzidine (TMB) under photo-irradiation, producing sensitive photo-catalyzed colorimetric response to SA. Under the optimal conditions, the proposed method could directly detect SA with the limit of detection (LOD) at 81 CFU mL^−1^ in PBS buffer in 5.5 hours, which demonstrated the sensitive and fast quantification of target pathogenic bacteria. The method showed weak colorimetric signal to *Escherichia coli* and *Pseudomonas aeruginosa*, indicating the high specificity for SA. In addition, the method can simultaneously detect 96 samples which can be used for high throughput analysis. The designed method may become a powerful tool for pathogenic microorganisms screening in clinical diagnostics, food safety and environmental monitoring.

## Introduction

*Staphylococcus aureus* (SA) is the most common pathogen that causes a wide range of human infections. It is the major cause of bacteremia, infective endocarditis as well as skin and soft tissue infection and device-related infections^1^. Rapid identification of SA in the early stages of infection is important for reducing high mortality. However, the conventional culture method, known as “gold standard” for bacterial detection, usually requires 3–5 days incubation. It also needs at least 12 hours of growth on solid media to complete the identification^2^. Time consuming and insensitive are common problems with these methods.

Various methods which can shorten the detection time and increase the sensitivity have been used for bacterial detection, including enzyme-linked immunosorbent assay (ELISA)^3^, polymerase chain reaction (PCR)^4^, surface plasmon resonance biosensor^5^, electrochemical biosensor^6^ and so on. Despite improvements, these methods still require sophisticated equipment, complex sample preparation and long-term blood culture, which limit their use in clinical applications^7^. Besides, false positive results often occur in PCR detection^8^. Therefore, the development of a new platform that can distinguish SA in a short time is highly desired.

In this study, an aptamer-based high throughput colorimetric biosensor for detection of SA was devised. The SA specific aptamer, reported by Y.S. Xu *et al*.^2^ has strong binding affinity to the bacteria. The TWJ DNA nanostructure used in the assay for signal amplification is another high light of the study. In recent years, as an enzyme-free amplification method, more and more attention has been paid to the self-assembly of nanostructures, which are molecules spontaneously combine into stable, well-defined aggregates under equilibrium conditions^9^. Among them, TWJ composed of three complementary oligonucleotide branches has become an extremely important building block for constructing DNA structure and dynamic assembly. Meanwhile, the TWJ strategy has obvious advantages, including simple probe design, economical biosensor manufacturing and excellent signal amplification. Therefore, it opens broad prospects for applications in biosensing and bioanalysis^10,11^.

X. F. Zhang etal reported a new discovery that the dsDNA-SG I complex possessed photocatalytic activity, which could catalyze the oxidation of oxidase substrates TMB with dissolved oxygen under photo-irradiation^12^. Compared with G-quadruplex-based DNAzyme^13,14^, peroxidase mimicking nanozyme^15^, and HRP^16,17^, which also have similar catalytic activity for TMB oxidation, the dsDNA-SG I proposed here offers several distinct advantages, such as simple and universal, label-free, visual sensing, suitable for convenient biosensor designs, etc.

In this study, for the first time, we designed an aptamer-based photo-irradiation colorimetric biosensor for detection of SA.By using this method, a new platform which can distinguish SA in a short time with selectivity and sensitivity has been achieved. The TWJ DNA nanostructure exhibited excellent signal amplification effect through self-assembly, and the dsDNA-SG I complex possessed photocatalytic activity, which made the method more specific and simpler. In addition, this analysis is a high throughput analysis which can be used to detect 96 samples at once. Compared with other reported methods, our biosensor has such advantages, include fast speed and more sensitive and specific. So, it may be a powerful tool for SA screening in clinical diagnostics.

## Experimental

### Reagents and apparatus

DNA oligonucleotides were synthesized and purified by Sangon Inc. (Shanghai, China). Their sequences are listed in Table 1. Streptavidin, bovine serum albumin (BSA), SYBR Green I (SG I), TMB, citric acid, disodium hydrogen phosphate, TE buffer, 0.1 M PBS buffer, 2 × SSC hybridization buffer and transparent ELISA plate were also purchased from Sangon Inc. (Shanghai, China). Colorimetric bioassay was carried out on a PHOMO Automatic enzyme immunoassay analyzer (Zhengzhou Antu Instruments Co. Ltd., China)

**Table 1:** Oligonucleotides used in the present work.

Oligonucleotides	Sequences (5′-3′)
SA aptamer:	TCCCTACGGCGCTAACCCCCCCAGTCCGTCCTCCCAGCCTCACACCGCCACCGTGCTACAAC
Capture probe:	bio-TTTTTGTTGTAGCAC
detection probe P1:	CTCATGGAGAGAGAATTTGGGTGCGAGACGTGCTACAA
detection probe P2:	CAGCGATCAGTTCAACTCTCTCCATGAG
detection probe P3:	GTCTCGCACCCAAAGAACTGATCGCTG

### **Preparation of*staphylococcus aureus*sample**

The strains of SA were cultured with Luria-Bertani medium at 37 °C for 24 hours, and then we diluted the SA suspension using 0.1 M PBS buffer to obtain an appropriate optical density value of about 0.5 at 600 nm. After that, the bacterial solution was serially diluted and inoculated onto the solid medium for 24 hours at 37 °C to quantify the CFU mL^−1^. The concentration was estimated by calculating the average number of CFU.

### Streptavidin coated transparent ELISA plate

Streptavidin was diluted in the coating buffer (100 mM Na_2_HPO_4_, 50 mM citric acid, pH 5.0) to the final concentration 5.0 µg mL^−1^. Then add 200 µL of the coating solution to each well. The plates were closed in a humidified box and incubated at 35 °C overnight.

Then the plates were washed with 0.01 M PBS buffer containing 0.05% Tween-20. After washing, 200 µL 0.1% bovine serum albumin PBS solution was added per well. The plates were saturated overnight at room temperature, then washed with 0.01 M PBS buffer containing 0.05% Tween-20 and dried at room temperature. Finally, the plate was filled with moisture absorbent and dried at 4 °C for storage^18^.

### Preparation of colorimetric biosensor

100 μL of 10 μM biotin labeled capture probe was dropped on the streptavidin-coated plate surface and incubated 30 minutes at room. After washed with the SSC buffer, 100 μL of 10 μM SA aptamer was added in the plates and incubated at 37 °C for 30 minutes. The plates were further rinsed with the washing buffer and different concentrations of SA were added in the reaction system, and then incubated for 2 hours at 37 °C. After SSC buffer washed the plates, three detection probes P1, P2, P3 (50 μL, 20 μM) were added to combine with the capture probe. After incubated at 37 °C for 90 minutes, 30 μL SYBR Green I (1:100) was embed in the dsDNA for 30 minutes, and then washed with PBS buffer. At last, 50 μL TMB (200 mg/L) and 20 μL 10% H_2_O_2_ were dropped in the plates. The plates were irradiated with white LED for 30 minutes. 50 μL 2 M H_2_SO_4_ were added to stop the solution. The absorbance were measured by PHOMO Automatic enzyme immunoassay analyzer in 450 nm and 630 nm

## Results and discussion

### Design of the colorimetric biosensor

**Figure 1:**
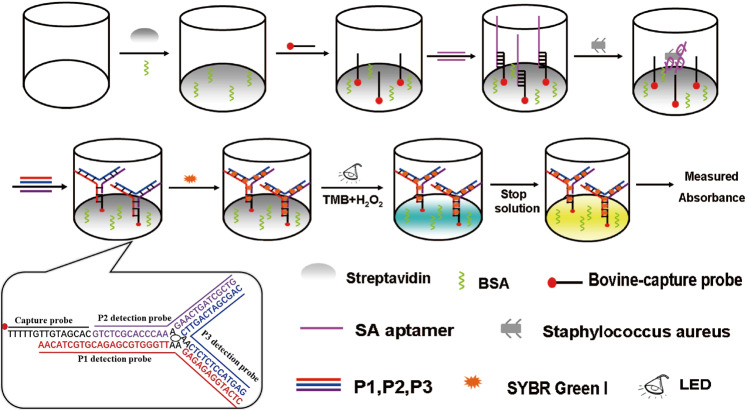
Schematic illustration of the principle for detection of *staphylococcus aureus* with aptamer-high throughput colorimetric biosensor based on photocatalytic activity of dsDNA-SG I complex.

An overview of the high throughput colorimetric biosensor for detection of SA based on TWJ DNA nanostructure and the catalysis of dsDNA-SG was illustrated in Fig. 1. The 96-well plate was coated with streptavidin. Then, BSA was used to avoid nonspecific adsorption on the plate surface. Biotin labeled capture probe anchored on the plate surface through streptavidin-biotin system. SA specific aptamer was immobilized on the 96-well plate by hybridization with the capture probe. In the presence of SA, for the stronger interaction between aptamer and SA, the aptamer was dissociated from the capture probe-aptamer duplex. Then the consequent single-strand capture probe could be hybridized with a TWJ DNA nanostructure which composed by three detection probes (P1, P2 and P3). After the addition of SG I, it formed dsDNA-SG I complex, which can catalyze the oxidation of TMB under LED photo-irradiation. The catalytic color is proportional to the added bacteria concentration. The designed biosensor showed acceptable precision and reproducibility. It provided a pragmatic tool for convenient detection of SA in clinical diagnosis.

### Signal amplification performance of designed biosensor

Compared to a single oligonucleotide branch, the TWJ nanostructure composed of three mutually complementary oligonucleotide branches (P1, P2, P3) can carry more SG I. Then the dsDNA-SG I complex catalyze oxidation of TMB under LED photo-irradiation. The catalytic color will further increase and the sensitivity will be improved. Meanwhile, the TWJ strategy also simplified probe design and biosensor fabrication, as well as excellent signal amplification. Thus, it opens a promising avenue for applications in biosensing and bioanalysis^10^.

### Optimization of experimental conditions

**Figure 2:**
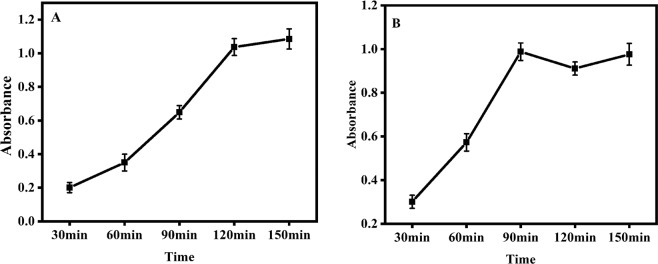
Optimizations of experimental parameters: (**A**) evaluation of binding time for specific aptamer and SA (**B**) evaluation of TWJ hybridization time for capture probe and P1/P2/P3 probe. (SA concentration: 10^4^ CFU mL^−1^).

In order to achieve the perfect assay performance, aptamer-SA incubation time and TWJ hybridization time were selectively optimized as the most valuable influence factors for the detection. Figure 2A showed the effect of aptamer-SA incubation time on the absorbance. The concentration of the test SA was 10^4^ CFU mL^−1^. With the increasing incubation time, the absorbance increased and tended to a steady value at 120 minutes. Prolonged incubation time did not increase the signal response obviously. So, 120 minutes was identified as the optimal incubation time. The effect of TWJ hybridization time for capture probe and P1/P2/P3 probe was also studied in the time range from 30 to 120 minutes. (Fig. 2B). The absorbance increased obviously within 90 minutes and then reached a platform. Thus, 90 minutes was selected as the appropriate time for the following experiments.

### Analytical performance of designed biosensor

**Figure 3:**
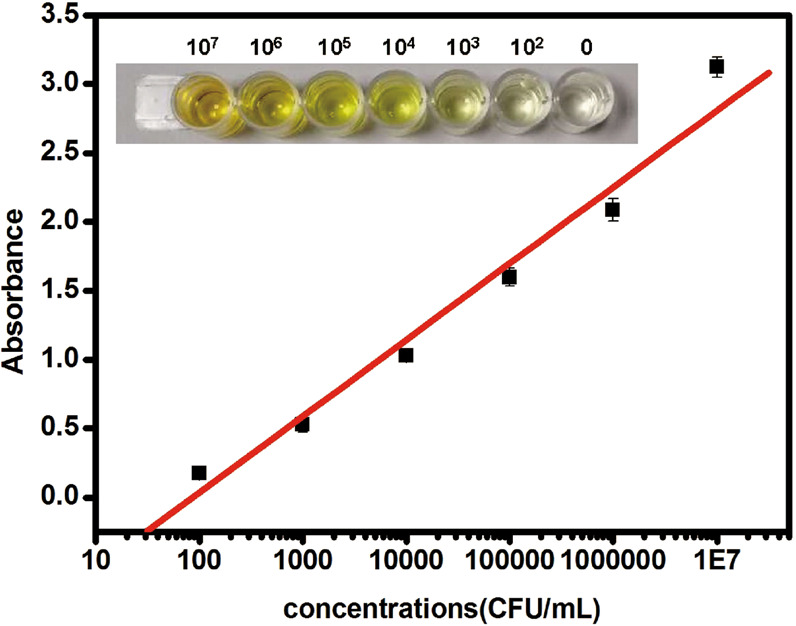
Absorbance responses to 10^2^, 10^3^, 10^4^, 10^5^, 10^6^, and 10^7^ CFU/mL *Staphylococcus aureus*.

The analytical performance of biosensor was performed under optimal experimental conditions. Serial dilution of SA in PBS buffer were to the final concentration of 10^2^, 10^3^, 10^4^, 10^5^, 10^6^, 10^7^ CFU mL^−1^. The absorbance increased with the increasing SA concentration. In the range of 10^2^ to 10^7^ CFU mL^−1^, the relationship between absorbance value and logarithm of SA concentration showed linear, and the correlation coefficient was 0.996 (Fig. 3). The detection limit was estimated to be 81 CFU mL^−1^ in PBS buffer. The biosensor proposed in this research can complete the detection of SA in 5.5 hours. Compared with conventional culture methods, this method is significantly time-saving and easier to operate^19,20^.

In order to further highlight the advantages of the designed biosensor in detecting SA, the analytical properties were compared with those of other biosensors^2,21,22^. Characteristics including the detection limit and dynamic range are summarized in Table 2. It proved that this method not only had great improvement in detection limit but also possessed the advantage of wide dynamic range.

**Table 2:** Comparison of analytical performance of the proposed method with other reported bioassays for the detection of *Staphylococcus aureus*.

Biosensor Platform	Bio-receptor	Detection limits CFU/mL	Dynamic range CFU/mL	Ref
Fluorescence	Aptamer	100	10^2^–10^4^	^2^
Fluorescence	Aptamer	150	3.4 × 10^2^–3.4 × 10^4^	^21^
Electrochemical	Aptamer	800	2.4 × 10^3^–2 × 10^4^	^22^
Colorimetric	Aptamer	81	10^2^–10^7^	This work

### Specificity of the strategy

**Figure 4:**
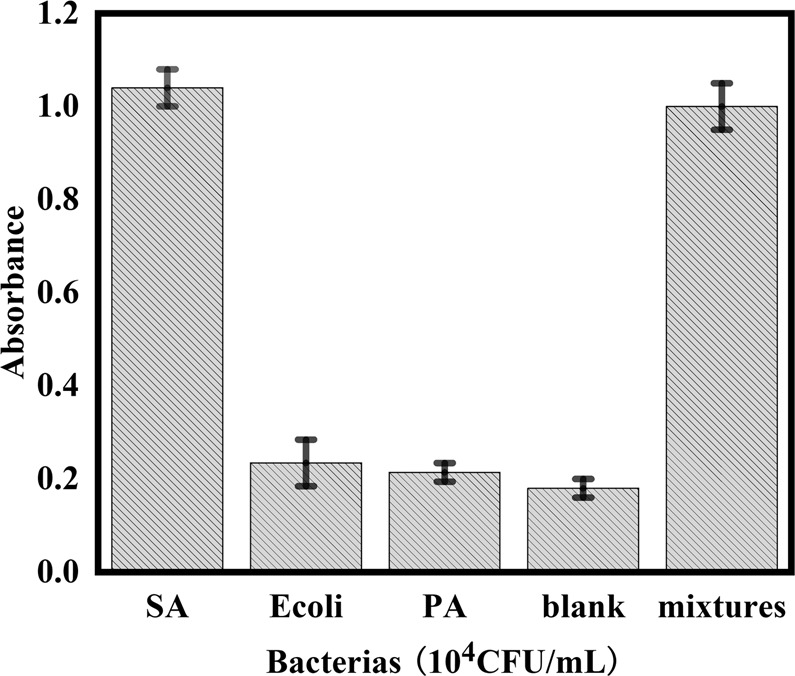
Absorbance responses of the colorimetric biosensor to 10^4^ CFU/mL *Staphylococcus aureus, Escherichia coli, Pseudomonas aeruginosa, microorganisms mixtures* and blank.

To prove the specificity of the biosensor, two bacterias, *Escherichia coli* (*E. coli*) *and Pseudomonas aeruginosa* (PA) were tested under the same experimental conditions as those for SA. The concentrations of the test bacteria were 10^4^ CFU mL^−1^. To further prove the identification ability of this method, mixtures of microorganisms (SA, PA, *E. coli*, 10^4^ CFU mL^−1^) were also tested in the meantime. Each bacterium and the mixtures of microorganisms were tested for three times. The results were shown in Fig. 4. The absorbance of these two bacteria (PA and *E. coli*) were only as low as the blank, but the SA and microorganisms’ mixtures had obvious absorbance responses, indicating that the colorimetric biosensor had a good specificity and identification ability for the detection of SA.

### Detection of SA in milk samples

**Figure 5:**
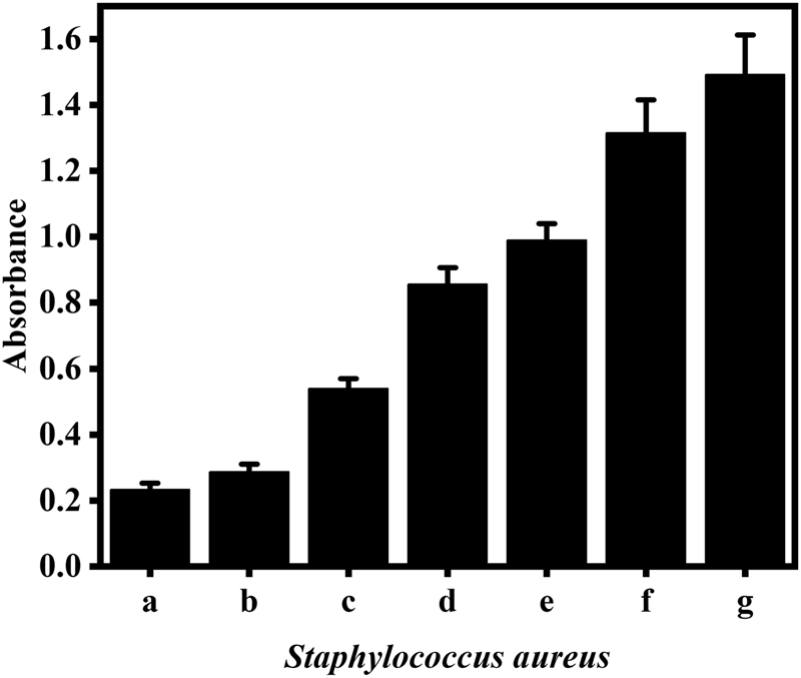
Absorbance responses to SA in the milk. 0 (**a**); 10^2^ (**b**); 10^3^ (**c**); 10^4^ (**d**); 10^5^ (**e**); 10^6^ (**f**); 10^7^ (**g**) CFU/mL SA.

In order to prove the proposed colorimetric biosensor can detect SA in actual samples sensitively and specifically, the cultured SA were inoculated into milk at a concentration of 0 to 10^7^ CFU mL^−1^. Each concentration was tested for three times. The absorbance responses of the biosensor to different SA concentrations were shown in Fig. 5. The responses increased with the increasing SA concentration. The fabricated bioassay method can detect SA concentration down to 100 CFU mL^−1^ in real milk samples. In addition, the method can complete the detection in 5.5 hours. Compared with traditional culture methods (3–5 days), this method is simple, fast, with higher sensitivity and specificity, showing the potential as a pragmatic tool for SA detection in real samples.

## Conclusions

Aptamer-based high throughput colorimetric biosensor for direct detection of *Staphylococcus aureus* has been successfully developed. The biosensor shows wide linear range, low detection limit, high specificity, and could be used for detection of SA in real samples. Importantly, the application of TWJ DNA nanostructure and photo catalytic activity of dsDNA–SG I complex not only amplifying the detection signal, but also shortening the detection time. The method provided a direct sensing platform for detection of SA and the whole analytical process can be finished in 5.5 hours. The bioassay strategy could be used to develop other assay method for pathogenic bacteria and would become a powerful tool for pathogenic microorganism screening in clinical diagnostics, food safety, biothreat detection and environmental monitoring.
